# Experimental Characterization of the Properties of Double-Lap Needled and Hybrid Joints of Carbon/Epoxy Composites

**DOI:** 10.3390/ma8115410

**Published:** 2015-11-11

**Authors:** A. Arnautov, A. Nasibullins, V. Gribniak, I. Blumbergs, M. Hauka

**Affiliations:** 1 Institute of Polymer Mechanics, University of Latvia, Aizkraukles Str. 23, Riga LV-1006, Latvia; nasib@inbox.lv (A.N.); Viktor.Gribniak@vgtu.lt (V.G.); ilmars-b@inbox.lv (I.B.); maris.hauka@gmail.com (M.H.); 2 Research Laboratory of Innovative Building Structures, Vilnius Gediminas Technical University (VGTU), Sauletekio Av. 11, Vilnius LT-10223, Lithuania

**Keywords:** carbon/epoxy composite, mechanical properties, joints, z-pins, hybrid

## Abstract

The effect of through-thickness reinforcement by thin 1 mm steel needles (z-pins) on the static tensile strength of double-lap joints of a carbon/epoxy composite was investigated. Two types of joints—z-pinned and hybrid (including glued ones)—were considered. The joints were reinforced in the overlap region with 9, 25, or 36 z-pins. Comparing mechanical properties of the double-lap joints with the corresponding characteristics of their unpinned counterparts, the z-pins were found to be highly effective: the strength and stiffness of the pinned joints increased up to 300% and 280%, respectively. These improvements were due to a transition in the failure mechanism from debonding of the joint in the absence of z-pins to pullout or shear rupture of z-pins or to the tensile failure of laminate adherends, depending on the volume content of the pins.

## 1. Introduction

Over the past three decades, the application of composite materials has been increasing continuously from traditional areas (such as aircraft engineering) to various fields in the automobile industry and civil engineering. There are two basic types of joints of composite structural elements: adhesive and mechanical. To realize the potential of advanced composites, especially in lightweight structures, such as aircrafts, it is important to ensure that the adhesively bonded or mechanically fastened joints do not reduce the effectiveness (in terms of the strength of materials and stiffness of the joints) of the structure and be lighter in weight than bolted joints [1]. Mechanical connections remain the key means for the transfer of loads between structural elements made of composite materials. Typically, mechanically fastened joints are made by means of rivets or bolts. In designing the joints, the reduced strength of composite materials to bearing capacity, their low elongation, brittle behavior (for example, carbon composites), creep and stress relaxation must be taken into account.

Holes in materials create stress or strain concentrations and hence reduce their strength [2]. Lekhnitskii [3] assessed the theoretical magnitude of stress concentration in composite material at the edge of a hole. For a plate loaded in tension, the stress concentration factor is calculated by the formula:

(1) $$k=1+\sqrt{2\left(\sqrt{\frac{E_{1}}{E_{2}}}-\nu_{12}\right)+\frac{E_{1}}{G_{12}}}$$

where *E*_1_ is the Young’s modulus in the direction of load axis; *E*_2_ is the Young’s modulus in the normal direction; *G*_12_ is the in-plane shear modulus and *ν*_12_ is the principal Poisson’s ratio. It is possible to increase strength of the connection by optimizing structure of a connected composite laminate. Experimental [4] and theoretical results [3] revealed that the best tensile performance is achieved using about 30% to 50% of ±45º plies in a laminate.

The stress concentration factor (Equation (1)) increases with the size of the hole [3,5]. Whitney and Nuismer [2,6] developed two criteria for the effect of holes on the tensile strength of composite laminates. They are commonly referred to as the Average Stress Criterion (ASC) and the Point Stress Criterion (PSC). Callus [4] reported that needles of a diameter ≤1.0 mm distributed at the distance greater than 3–5 diameters do not produce any weaknesses of material in the jointed composite sheets. Reduced diameter of the fasteners allows us to achieve a uniform distribution of stresses along the joints. This also eliminates the “cut-fiber” effect in the joints. To improve the ultimate strength of the joints, the bonded region is often reinforced with stitches [7,8,9,10]. Stitching with high strength-fibrous yarns in the overlap region increases the tensile strength of the joint up to 40% [11]. The stitching requires the sewing of a continuous high-strength reinforcing thread through an uncured pre-preg laminate or pre-form fabric before the consolidation or impregnation phase [10,12]. Numerous investigations, ranging from analytical studies [13,14,15] to numerical (finite element) analyses [7,16,17], have been conducted to identify the mechanics of stitching or z-pinned reinforcement. The bridging mechanism of stitches and z-pins was extensively discussed in the literature [14,15].

Most of the studies (e.g., [7,8,9,10,11,12,13,14,15,16,17,18,19,20]) were focused on the benefits of z-pinned laminates such as improved delamination toughness, impact damage tolerance, and joint strength. Although these studies contributed to the clarification of the interaction between stitches and laminate, up to now, the effect of various stitching parameters on the fracture performance of lap joints has not been adequately understood. The adverse effects of z-pinned joints, particularly reductions to the in-plane mechanical properties [21], have received less attention from researchers.

The main disadvantages of the stitched laminates can be related to the reductions in in-plane mechanical properties (especially in pre-preg laminates) due to a failure and misalignment of the fibers and increased concentration of the resin (polymer) around the needles [12]. Furthermore, the bond-line thickness increases up to 20% with the pin content due to the swelling of the pre-preg stack to accommodate the pins [11]. The increase in thickness can alter the magnitude of the bond-line shear stress induced in the joint. Moreover, most of the pins become misaligned during the insertion and subsequent autoclave consolidation of the lap joints [11].

The current manuscript presents an alternative approach for improving the ultimate strength of the lapped joints. Unlike in previous works that consider the lap joints reinforced with stitches before consolidation of the adherends, thin steel needles reinforce the bonded region after the consolidation. The new pinning technique allows us to avoid concentration of the resin-rich zones around the pins, securing homogeneous distribution of the polymer within the adherends. Moreover, it eliminates misalignment of the fibers mentioned in the reference [12]. Damages of the adherends (the “cut-fiber” effect) are reduced with decreasing diameter of the pins. The corresponding effects (improved mechanical properties) can be considered as the main advantage of the proposed pinning technique in respect to the traditional stitching technologies. The tensile behavior of double-lap joints z-pinned with thin steel needles of diameter 1 mm is the object of the present experimental study. The effect of the content of the z-pins on the strength of carbon/epoxy composite joints is investigated. The adhesive joint is chosen as the reference evaluating effectiveness of two alternative types of overlap connections: purely pinned and hybrid (additionally glued) joints.

## 2. Materials and Experimental Technique

Double-lap joints (DLJ) of a carbon fiber epoxy laminate with a [0°/90°/±45°]_s_ lay-up were investigated. Test specimens were cut from plates with the (0°) fibers along the joint length, which was also the direction of tensile loading. The mechanical properties of the carbon fibers reinforced plastic plate are shown in Table 1. The overlap region was 30 mm long and 30 mm wide. The joints were pinned through the overlap region by using 16 mm long needles made of carbon steel S185 (Erkrath, Germany). The mechanical properties of the needles are shown Table 2.

**Table 1:** Tensile properties of carbon fiber epoxy laminate plates (of thickness 3.5 mm).

Young’s Modulus, E_11_, GPa	Poisson’s Ratio *ν*_21_	Poisson’s Ratio *ν*_31_	Ultimate Tensile Strength σ_11,_ MPa
76.7 ± 0.8	0.44 ± 0.03	0.042 ± 0.003	520 ± 26

**Table 2:** Mechanical properties of steel needles.

Tensile Modulus of Elasticity, GPa	Tensile Yield Point, MPa	Ultimate Elongation, %	Poisson’s Ratio *ν*	Shear Modulus, GPa
200	420	18	0.31	80.0

**Figure 1:**
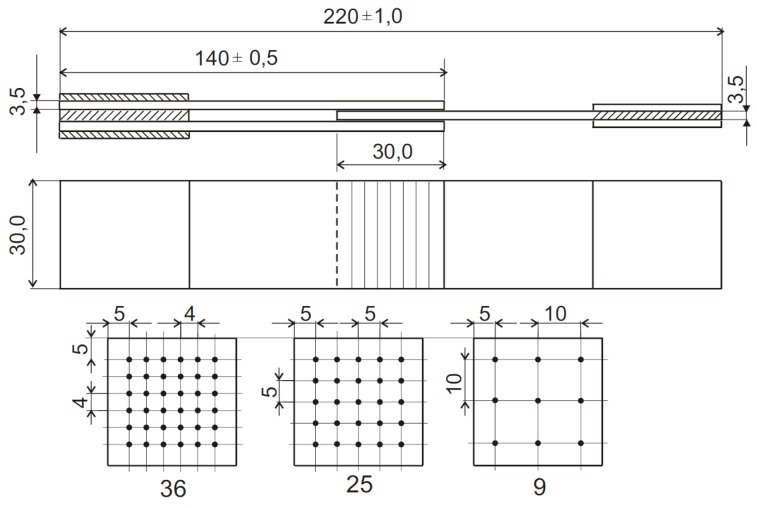
Geometry of the samples and distribution schemes of the pins (all units in mm).

The dimensions and the geometry of the samples are shown in Figure 1. Two types of DLJ were investigated: only pinned with needles and hybrid (needled and adhesively bonded). For a reference, adhesive DLJ (without pins) were also made. The epoxy compound Sikadur^®^ 52 was used for production of the adhesive DLJ. The mechanical properties of the adhesive are given in Table 3.

**Table 3:** Mechanical properties of Sikadur^®^ 52 adhesive [22].

Tensile Modulus, GPa	Tensile Strength, MPa	Ultimate Strain, %	Poisson’s Ratio *ν*
1.93	42.0	3	0.31

**Figure 2:**
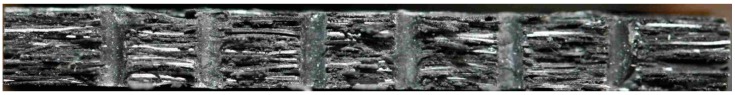
Side-view on drilled holes in the overlap region.

**Figure 3:**
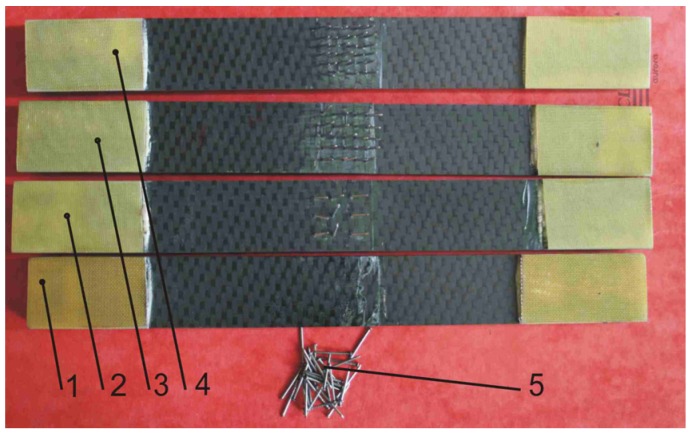
Double-lap adhesive joint (1) and hybrid joints with 9 (2), 25 (3) and 36 (4) pins, and the needles (5).

The joints were produced using 9, 25, and 36 pins. As shown in Figure 2, the pins were inserted (pinch-fitted) into holes of diameter 1.0 mm drilled through an overlap region (Figure 1). The considered configurations of DLJ are shown in Figure 3.

**Figure 4:**
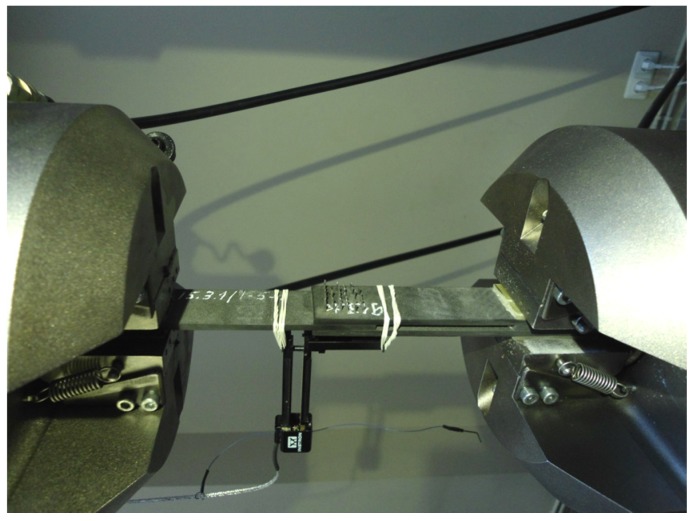
The tensile test setup.

To determine the apparent strength of the joint, shear tests were performed according to ASTM D 3983. The specimens were tested using MTS 809.40 (MTS Systems Corp., Eden Prairie, MN, USA) servo-hydraulic machine with 250 KN load cell. The test setup is shown in Figure 4. The ultimate strength and elongation of the joints were determined in monotonic loading at a crosshead speed of 2 mm/min. At least six specimens were tested for each type of DLJ.

The applied load-displacement diagrams were recorded during testing. The shear stresses were calculated dividing the applied tensile load by the total bond area. The ultimate shear stress, the strain at the maximum shear stress, and the shear stiffness of DLJ (in the range from 0% to 10% of the ultimate loads) were also calculated using the load-displacement recordings.

## 3. Results and Discussion

**Figure 5:**
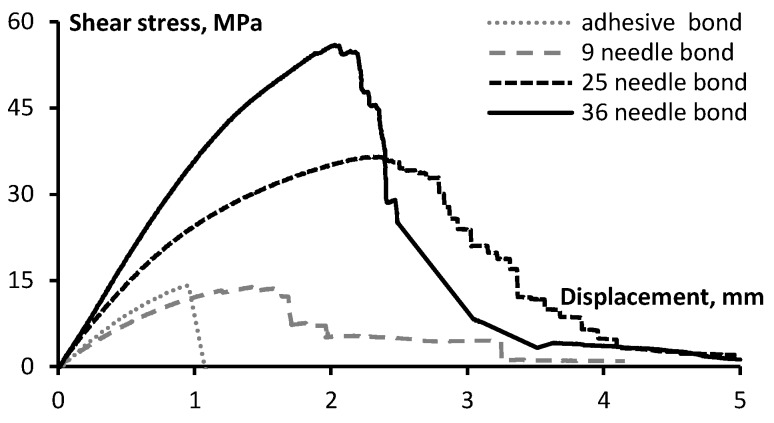
Shear stress-displacement diagrams of the double-lap needled joints reinforced with different numbers of the z-pins.

**Figure 6:**
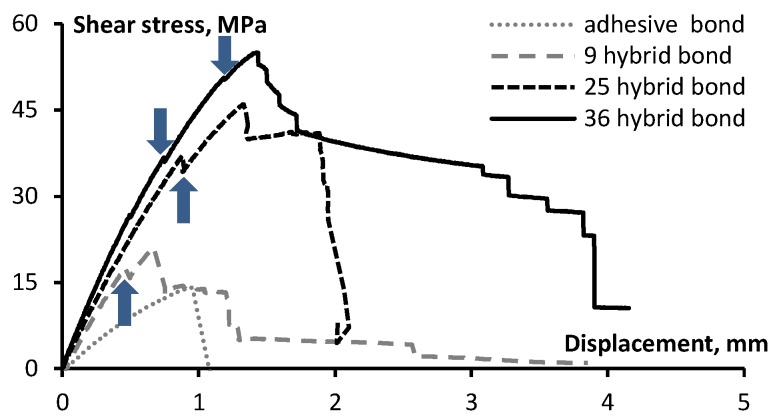
Shear stress-displacement diagrams determined for the adhesive double-lap joints with different numbers of the z-pins (arrows indicate the drop in the shear stress).

The tests were carried out to assess the effect of content (*i.e.*, number) of the z-pins on the strength and deformation properties of the joints. Figure 5 and Figure 6 show the averaged load-displacement diagrams of pinned-only and hybrid DLJ with a different content of the z-pins. As can be observed, the shear stiffness increases gradually with the number of the pins. The curve of the unpinned (adhesive) joint rises almost linearly up to the peak stress of 14.2 MPa afterwards failing in a brittle manner due to the bond delamination. The failure tends to be catastrophic, which is a characteristic of the epoxy resin (a rather brittle material). Similar results were observed for stitched joints [11].

**Figure 7:**
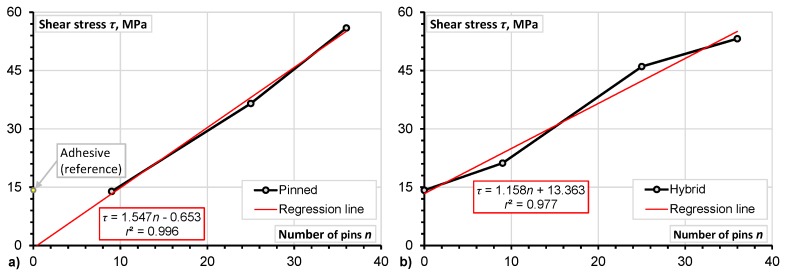
The z-pins effect on the ultimate shear stress of pinned-only (**a**) and hybrid (**b**) double-lap joints (DLJ).

**Figure 8:**
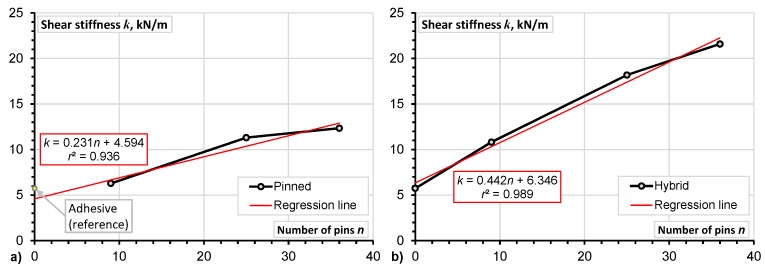
The z-pins effect on the shear stiffness of pinned-only (**a**) and hybrid (**b**) DLJ.

Diagrams of the pinned (Figure 5) and the hybrid joints (Figure 6) initially increase in the same way as those of the adhesive joint, though possessing higher stiffness and strength. For the hybrid joints, a small drop in the diagram occurs (at deformations between 0.4 and 1.5 mm, see Figure 6) before the load increases up to the ultimate value. Such a drop corresponds to the opening of a debonding crack (without mechanical destruction of the joint). The pins, bridging the cracks, resist the applied load. For the pinned joint (Figure 5), the drop in the load was not observed; furthermore, the ultimate load bearing capacity corresponded to the elongation greater than it was achieved in the hybrid DLJ. The test results are summarized in Table 4 and Figure 7 and Figure 8. The following observations can be made to compare deformation behavior of DLJ:

- Improvement of the mechanical properties of DLJ is significantly correlated with the number of pins (the minimum value of the determination coefficient *r*^2^ is equal to 0.94).
- Application of z-pins improved the mechanical properties of DLJ. As can be observed from Table 4, the strength and the stiffness (calculated in the range from 0% to 10% of the ultimate load) of the pinned-only joints was increased by 300% and 120%, respectively. For the hybrid joints, the increase in both strength and stiffness was the same (*i.e.*, 280%).
- Comparing the mechanical properties of the reference (adhesive) and pinned-only joints (Figure 7a and Figure 8a), the extrapolated regression predictions clarify the tendency for the number of pins to increase the strength; for the shear stiffness, this effect is less evident.
- Increment in the shear strength related to the number of pins is less significant in the hybrid DLJ in comparison with the pinned ones (compare the slope coefficients of the regression line, which are equal to 1.547 and 1.158 for the hybrid and pinned joints, respectively). Considering the shear stiffness, the opposite tendency can be evidenced from Figure 8. For instance, the shear stiffness of the hybrid DLJ with 36 pins is 1.7 times higher than that of a similar pinned joint (Table 4). Most probably, this effect is the consequence of the effective composite action between z-pins and adhesive achieved in the proposed hybrid DLJ.
- The hybrid connection reduces deformability of the joint – the elongation corresponding to the maximum load of the pinned DLJ was twice the hybrid counterpart one (Table 4).

**Table 4:** Effect of the number of z-pins on the mechanical properties of double-lap joints.

Joint Type	Number of z-Pins	Ultimate Shear Strength $\tau_{ult}$, MPa	Elongation at the Ultimate Load, mm	Shear Stiffness, kN/m
Adhesive (reference)	0	14.2 ± 2.5	0.95 ± 0.09	5.7 ± 0.6
Needled bond	9	13.9 ± 3.2	1.42 ± 0.25	6.3 ± 0.7
Needled bond	25	36.5 ± 5.1	2.31 ± 0.28	11.3 ± 0.9
Needled bond	36	55.9 ± 6.2	2.03 ± 0.29	12.3 ± 0.7
Hybrid bond	9	21.2 ± 2.4	0.67 ± 0.19	10.8 ± 0.6
Hybrid bond	25	46.0 ± 2.9	1.34 ± 0.31	18.2 ± 0.8
Hybrid bond	36	53.7 ± 3.3	1.56 ± 0.31	21.6 ± 1.2

**Figure 9:**
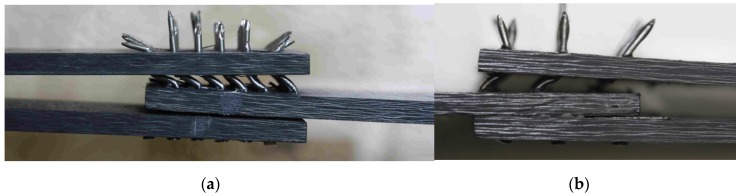
Failed hybrid joints: DLJ with 36 z-pins (**a**) and DLJ with 9 z-pins (**b**).

**Figure 10:**
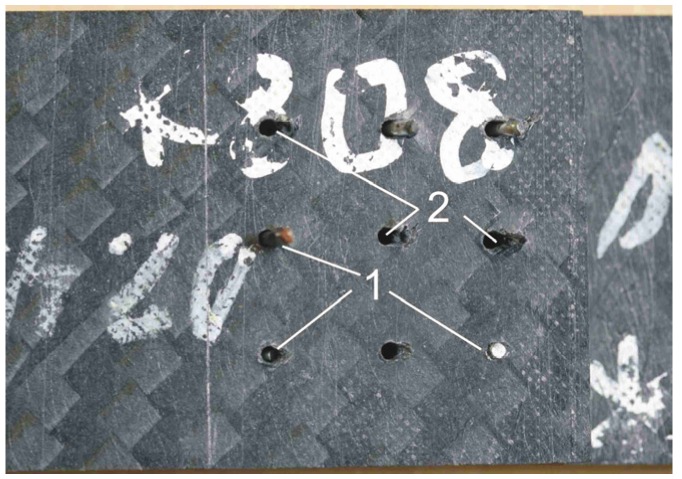
Different behavior of the pins within the failed hybrid DLJ: transverse shear failure (1) and bearing failure (2).

Figure 9 and Figure 10 illustrate failure mechanism of the selected hybrid DLJ. It can be observed that the local bending of the pins was the cause of the failure of DLJ with 36 z-pins (Figure 9a). Reduction of the number of pins results in shear failure of some of them (Figure 9b and Figure 10). Independently of the presence of additional adhesive connection, such a failure mechanism was characteristic of all of the pinned joints. Due to the loss of the adhesive connection, the failure of the reference joints was brittle. The observed bridging effect of the z-pins (Figure 9), transferring the shear stresses through the crack, is the general benefit of the pinned DLJ compared to the reference ones.

## 4. Conclusions

A new pinning technique of double-lap joints (DLJ) has been proposed. It is based on application of thin steel needles of 1 mm diameter (z-pins) as through-thickness reinforcement of a double-lap joint. The tensile behavior of double-lap joints of a carbon fiber epoxy laminate was experimentally investigated. Two types of DLJ were considered: purely pinned and hybrid (additionally glued) joints. The joints were produced using 9, 25, and 36 pins. For the reference, adhesive DLJ (without pins) were also made. The obtained results revealed that:

- Improvement of the mechanical properties of DLJ is significantly correlated with the number of z-pins: the strength and stiffness (calculated in the range from 0% to 10% of the ultimate load) of DLJ increased up to 300% and 280%, respectively.
- Increment in the shear strength related to the number of pins is less significant in the hybrid DLJ compared to the pinned ones. However, the opposite tendency was evidenced considering the shear stiffness of the joints. The shear stiffness of the hybrid DLJ with 36 pins was found to be 1.7 times higher than that of a similar pinned joint.
- The hybrid connection reduces deformability of the joint—the elongation corresponding to the maximum load of the pinned DLJ was twice that of the hybrid counterpart.
- The observed bridging effect of the z-pins, transferring the shear stresses through the crack, is the general benefit of the pinned DLJ compared with the reference adhesive joints, the failure of which was their brittleness.
